# The Impact of Different Periods of Walking Experience on Kinematic Gait Parameters in Toddlers

**DOI:** 10.3390/ijerph19010058

**Published:** 2021-12-22

**Authors:** Marta Gimunová, Martin Sebera, Michal Bozděch, Kateřina Kolářová, Tomáš Vodička, Martin Zvonař

**Affiliations:** 1 Department of Kinesiology, Faculty of Sports Studies, Masaryk University, 62500 Brno, Czech Republic; sebera@fsps.muni.cz (M.S.); michal.bozdech@fsps.muni.cz (M.B.); tvodicka@fsps.muni.cz (T.V.); zvonar@fsps.muni.cz (M.Z.); 2 University Sport Centre, Faculty of Sports Studies, Masaryk University, 62500 Brno, Czech Republic; katerina.kolarova@fsps.muni.cz

**Keywords:** gait, children, motor development, temporal-spatial parameters, kinematic analysis

## Abstract

This study aimed to analyse the kinematic differences in gait between three groups of toddlers who differed in their weeks of independent walking (IW) experience, but not in anthropometrical characteristics, to determine the relationship between walking experience without the side effect of morphological differences on gait parameters. Twenty-six toddlers participated in this study. Depending on the week of their IW, toddlers were divided into three groups: Group 1 (1–5 weeks of IW), Group 2 (6–10 weeks of IW), and Group 3 (11–15 weeks of IW). Each toddler walked barefooted over a 2-m long pathway, and 3D kinematic data were obtained. A decrease in the upper limb position, hip flexion, and step width, i.e., changes towards the adult gait pattern, were observed in Group 3. Less experienced walkers exhibited a wider step width despite no statistically significant difference in body mass and height between groups. Results of this study show no statistically significant difference in step length between groups, suggesting that step length is more related to height than to the walking experience. The increased step length in more experienced walkers reported in previous studies may therefore be a result of different heights and not walking experience.

## 1. Introduction

The development of walking in toddlers is an important kinematic milestone. Walking patterns can vary widely amongst toddlers and are characterised by unique biomechanical strategies [1]. Walking alone was reported to have the widest variety in the age of achievement compared to other gross motor developmental milestones in babies and toddlers [2].

Toddlers perform their first unsupported steps between approximately 8.2 to 17.6 months of age (estimated 1st and 99th percentile) [2,3] and are gradually refined with practice and maturity [2]. To be able to walk, the toddler must acquire postural stability control with respect to gravity. Pre-walking toddlers at about 10 months of age can maintain the static equilibrium; however, they perform a greater centre of pressure sway compared to adults [4]. This greater disequilibrium, measured by the distance of the centre of mass to the centre of pressure, was observed in toddlers at the onset of walking [5]. Furthermore, toddlers must acquire dynamic control of the body to perform forward motion. The swing phase, when only one leg supports the whole body, is the most challenging problem when learning to walk [6,7,8]. Walking maturation consists of two phases. During the first phase, up to six months after independent walking (IW), the dynamic equilibrium and trunk stabilisation during gait improves, the second phase (up to 7 years) is characterised by a more precise adjustment of the gait parameters [9,10].

The early stage of IW is characterised by large individual variability in walking maturation strategies. There are some typical patterns in this variation within this early stage of walking. In novice walkers with less than 22 weeks of walking experience, three different patterns of foot contact were described: (i) heel strike (characterised by the initial contact of heel and then the heel to toe roll-over process continues), (ii) flatfoot contact (characterised by simultaneous contact of the forefoot and heel and subsequent heel to toe roll-over), and (iii) forefoot contact (characterised by the initial contact of metatarsals, then the midfoot and heel are placed on the ground and subsequent heel to toe roll-over follows) [11,12]. In toddlers, a wide base of support, asymmetrical foot rotation, and high foot lift during the swing phase of the step cycle were observed compared to the adult gait [13,14].

The classic pendulum mechanism is not yet implemented in novice walkers [14], it develops after a few months of walking experience, and it is evident in improved walking patterns [15] by adopting more effective solutions [16]. Still, little is known about changes in the gait pattern in the early phases of IW in relation to the walking experience. Understanding gait development changes in this period can be essential for the explanation of different solutions of IW.

Previous studies focused on the early stages of walking without considering the effect of different body masses, heights, and the age of their participants. It can be hypothesised that some of the changes associated with walking experience in these studies might instead reflect the body mass or height changes. In this study, the kinematic differences in gait between three groups of toddlers who, despite no statistical difference in age (as the inclusion criteria consisted of age as closed to 17 months as possible), body mass, and height, differed in their weeks of walking experience were analysed. The purpose of this study was to assess the differences in kinematic gait parameters related to walking practice to gain a better understanding of the ontogenetic development of human walking.

## 2. Materials and Methods

### 2.1. Participants

Originally, 30 healthy toddlers participated in this study; however, 4 toddlers from Group 1 (one to five weeks of IW) were not able to cooperate during the data collection. Data of 26 healthy toddlers of a similar age, height, and body mass were included in this study. Inclusion criteria consisted of typical development, an age as close to 17 months as possible, and gestational age of more than 37 weeks. The exclusion criteria consisted of any foot or lower limb deformities and any significant previous foot or lower limb injuries or operations. Depending on the week of their walking experience defined by the first five consecutive steps without any support reported by parents, participants were divided into three groups: Group 1 (*n* = 6) had one to five weeks of walking experience, Group 2 (*n* = 10) had six to ten weeks of walking experience, and Group 3 (*n* = 10) had eleven to fifteen weeks of walking experience. Informed consent was provided by legal representatives of the toddlers prior to measurement. This study was approved by the local Research Ethics Committee. The measurement was performed in accordance with the ethical standards of the Helsinki Declaration.

### 2.2. Procedures and Equipment

Each participant was encouraged to walk several times to obtain five attempts with no unexpected change of direction or fall, barefooted over a 2-m long pathway towards a parent or a toy at a self-selected speed, wearing a bodysuit or a diaper as the clothes were observed to modify the walking pattern in toddlers [17]. One gait cycle of each participant during the third attempt, if no unexpected change of direction or fall occurred, was used for further analysis.

**Figure 1:**
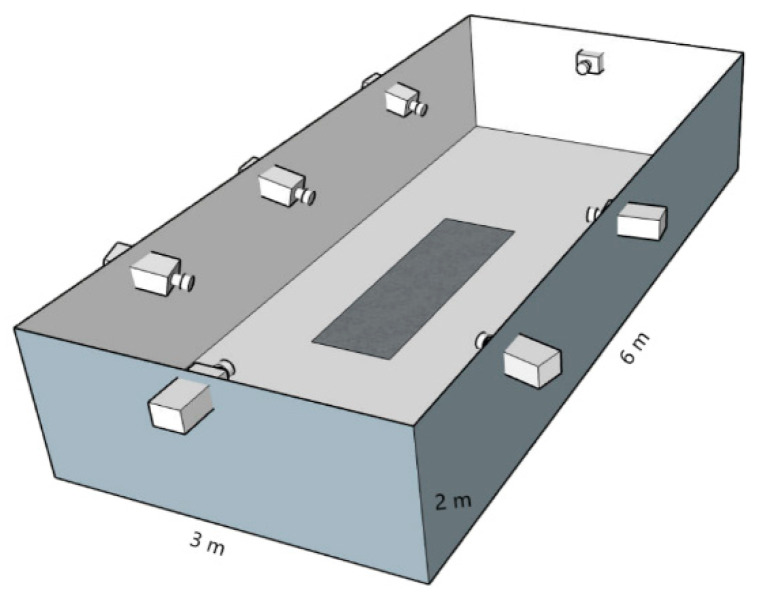
Camera placement during the 3D kinematic data collection.

The gait 3D kinematic data were obtained using seven cameras (Basler A602fc, Unterschleißheim, Germany) from the Simi Motion System, filmed at 100 Hz. The camera placement with respect to the walking line is shown in Figure 1. Selected anthropometric points were tracked using the Simi Motion Software: left and right *acromiale, iliospinale anterius, tibiale laterale, malleolus lateralis,* and *stylion*. The beginning of the gait cycle was determined by the first foot contact with the ground.

The following parameters were used for further analysis. For the angular parameters of the hip and knee joint range of motion (ROM), the maximum and minimum for the right and left sides were analysed. The maximal angle was analysed for upper limb position, for anterio-posterior and medio-lateral trunk lean, the ROM was analysed.

- stride time (s): time between foot contact and subsequent foot contact of the same foot.
- step length (m): distance between consecutive steps.
- step width (m): distance between the left and right *malleolus lateralis* (ankle spread).
- hip joint angle (°): angle between the point *acromiale, iliospinale anterius,* and *tibiale laterale* in the sagittal plane during the gait cycle.
- knee joint angle (°): angle between the point *iliospinale anterius, tibiale laterale,* and *malleolus lateralis* in the sagittal plane during the gait cycle.
- upper limb position (°): angle between the point *iliospinale anterius, acromiale,* and *stylion* during the gait cycle.
- anterio-posterior trunk lean (°): angle between the point *acromiale, iliospinale anterius,* and sagittal plane during the gait cycle.
- medio-lateral trunk lean (°): angle between the point *acromiale, iliospinale anterius,* and frontal plane during the gait cycle.

### 2.3. Statistical Analysis

Most of the variables did not meet the assumptions of normal distribution and homogeneity of variance, verified by a test of normality (Shapiro–Wilk test) and Levene test of homogeneity of variances. Therefore, a Kruskal–Wallis test and multiple comparisons *p* values were used to compare the differences between groups together with eta^2^ (effect size for Kruskal–Wallis test, eta^2^ = (H − k + 1)/(n − k); where H is the value obtained in the Kruskal–Wallis test; k is the number of groups; n is the total number of observations). The thresholds values according to the Cohen [18] rules of thumb were set as small (eta^2^ = 0.01), moderate (eta^2^ = 0.06), or large (eta^2^ = 0.14) effect.

*p* ˂ 0.05 was considered statistically significant. The statistics were obtained using the Statistica TIBCO Software Inc. (Palo Alto, CA, USA), version 13.5.

## 3. Results

A Kruskal–Wallis test and multiple comparisons of *p* values were used to compare the differences between Group 1, 2, and 3. No statistically significant differences between groups were observed in body mass (*p* = 0.379), height (*p* = 0.981), age (*p* = 0.211), and BMI (*p* = 0.178). The groups’ characteristics are shown in Table 1.

In Table 2, the Means and SD of the analysed gait parameters for each group are shown. 

When comparing groups, a statistically significant difference was observed in the minimum left hip joint angle between Groups 1 and 3 (*p* = 0.030 eta^2^ = 0.218), where an increase in this angle was observed in more experienced walkers. Statistically, significant differences were also observed in the left and right upper limb positions between Groups 1 and 3 (*p* = 0.026, eta^2^ = 0.230; *p* = 0.008, eta^2^ = 0.331, respectively), Group 1 demonstrated a more upward position (the so-called high guard position) of the upper limb. Step width statistically differed between Groups 1 and 3 (*p* = 0.005, eta^2^ = 0.369), where less experienced walkers exhibited wider step width. Differences in other parameters did not reach statistical significance. Group 2 did not differ with a statistical significance from Group 1 and Group 3.

## 4. Discussion

The aim of this study was to analyse the kinematic differences in gait between three groups of toddlers who differed in their weeks of experience with IW but not in their age, height, or body mass. Knowledge regarding the relationship between the walking experience without the side effect of body mass, height, and age on gait parameters is limited. Most of the previous studies analysed the early stages of walking in participants of different ages, heights, and body masses [15,19,20]. In a previous study by Van Dam et al. [21], it was suggested that the walking pattern in toddlers is significantly related to the morphology of the head and pelvis. On the other hand, in a previous study by Kingsnorth and Schmuckler [22], walking experience was the parameter most strongly related to the ability to cross a barrier, compared to body size or age.

A decrease in the left and right upper limb position between Group 1 and Group 3 was observed in this study, where Group 1 performed a more upward position (so-called high guard position) of the upper limb. A similar finding was reported in previous studies by Kubo and Ulrich [20] and Ledebt [19], who described the lowering of the arm position and then movements in the shoulder and elbow joints when the postural stability of the toddler during the gait becomes more stable. When the first steps are performed, the arm movement could challenge the stable posture during walking [19].

In adults, step width was observed to be affected mainly by gender [23] and body mass [24]. Similarly to the studies by Ledebt [19] and Ivanenko et al. [14], in this study, a statistically significant difference was observed in the step width between Group 1 and Group 3. Less experienced walkers exhibited a wider step width despite no statistically significant difference in body mass and height between groups. In addition, in a previous study, the walking experience was found to affect the step width more than the body length [21]. Additionally, a strong relationship was observed between narrowing step width and the decrease of arm guard posture [19], similarly to the observations of this study. The narrow step width is an advantage for the toddler as it reduces the metabolic cost of walking [21].

In previous studies, step length was reported to increase in more experienced walkers [25,26]. Results of this study show no statistically significant difference in step length between groups. In a study by Van Dam et al. [21], a stronger correlation of step length with body length than with the walking experience was observed. The increased step length in more experienced walkers reported in previous studies may therefore be a result of different heights and not the walking experience.

A statistically significant decrease in the left hip joint flexion was observed in this study in Group 3 compared to Group 1. The decrease in this angle was observed in more experienced walkers. To enhance postural stability when walking, hip extension, during which the hip remains slightly flexed, dominates during gait in toddlers. In a previous study comparing the gait of toddlers and adults, the hip flexion in adults was smaller [27], similar to the most experienced group of walkers in this study. 

The limitation of this study is the small number of participants and their division into groups 1, 2, and 3 by one to five, six to ten, and eleven to fifteen weeks of walking experience and not by months of walking experience as both beginners and experienced walkers were desired to be of the same chronological age. Compared with the normal variation in age of walking alone milestone achievement, all participants started their walking relatively late; approximately 80, 90, and 97 percentiles for Group 1, Group 2, and Group 3, respectively [2]. This probably reflects the different definition of the first unsupported steps (defined in this study as five consecutive steps without any support) or the cultural differences in the caretaking environment on the infants’ motor development [28]. Future studies focusing on the effect of the child development rate, e.g., through the time of achieving the gross motor developmental milestones such as standing alone or walking alone, and kinematic and dynamic gait parameters in toddlers will bring a more detailed insight into gait maturation.

## 5. Conclusions

To conclude, walking experience seems to be the main factor in gait parameter changes. A decrease in left and right upper limb position, hip flexion, and step width, i.e., changes towards the adult gait pattern, between the least (Group 1, one to five weeks of IW) and the most experienced group (Group 3, eleven to fifteen weeks of IW) of novice walkers was observed despite no statistically significant difference in their age, body mass or height between the groups. Toddlers with the middle walking experience (Group 2, six to ten weeks of IW) did not express a statistically significant difference from Group 1 and Group 3.

## Figures and Tables

**Table 1:** Groups’ characteristics.

		Body Mass (kg)	Body Height (cm)	Age (Months)	BMI	Walking Experience (Weeks)	Gender
Group 1	Mean	9.90	78.50	16.92	16.07	3.67	female *n* = 3
	SD	1.09	4.97	2.57	1.08	1.75	male *n* = 3
Group 2	Mean	10.15	77.95	15.79	16.75	7.80	female *n* = 5
	SD	0.99	3.13	1.70	1.88	1.14	male *n* = 5
Group 3	Mean	10.66	77.70	17.07	17.75	12.30	female *n* = 5
	SD	1.29	5.33	1.98	2.29	1.57	male *n* = 5
	*p*	0.379	0.981	0.211	0.178		

**Table 2:** Mean and SD of analysed parameters for Groups 1, 2, and 3.

		Group 1		Group 2		Group 3		*p*
		Mean	SD	Mean	SD	Mean	SD	
Temporo-spatial parameters	Stride time (s)	1.02	0.17	1.05	0.15	1.05	0.19	0.878
	Step length (cm)	0.18	0.03	0.18	0.05	0.18	0.05	0.704
	Step width (cm)	0.19	0.04	0.15	0.05	0.12	0.02	0.005 ^A^
Hip joint angle, left	RoM (°)	42.53	10.15	33.22	7.89	29.11	7.70	0.037 ^A^
	Min (°)	131.50	10.64	141.99	8.93	147.43	9.76	0.030 ^A^
	Max (°)	174.04	2.88	175.20	8.46	176.54	5.16	0.144
Hip joint angle, right	RoM (°)	40.46	7.91	32.28	8.59	33.35	8.76	0.206
	Min (°)	134.19	12.72	142.42	8.72	145.05	8.98	0.222
	Max (°)	174.65	8.73	174.70	5.54	178.39	1.22	0.285
Knee joint angle, left	RoM (°)	71.14	10.67	63.58	11.75	68.56	8.28	0.413
	Min (°)	103.99	9.08	107.94	8.00	105.56	10.85	0.781
	Max (°)	175.13	3.91	171.52	8.08	174.11	8.03	0.206
Knee joint angle, right	RoM v	67.40	12.52	63.78	7.21	71.05	15.52	0.472
	Min (°)	106.91	12.05	108.58	3.37	104.57	14.78	0.182
	Max (°)	174.31	5.41	172.36	7.26	175.61	4.32	0.627
Upper limb position, left	Max (°)	78.30	23.43	61.08	21.81	48.72	10.79	0.026 ^A^
Upper limb position, right	Max (°)	86.59	26.54	65.66	22.61	46.94	17.40	0.008 ^A^
Anterio-posterior trunk lean	RoM (°)	8.46	4.30	7.64	2.07	5.86	2.54	0.096
Medio-lateral trunk lean	RoM (°)	6.33	2.56	7.06	2.09	6.31	2.55	0.727

RoM—Range of Motion, ^A^ statistically significant difference between Group 1 and Group 3.

## Data Availability

The data presented in this study are available upon request from the corresponding author. The data are not publicly available due to ethical restrictions.
